# Molecular Identification of ten species of stored-product psocids through microarray method based on ITS2 rDNA

**DOI:** 10.1038/s41598-017-16888-z

**Published:** 2017-12-01

**Authors:** Li-Jun Liu, Ao-Han Pang, Shi-Qian Feng, Bing-Yi Cui, Zi-Hua Zhao, Zuzana Kučerová, Václav Stejskal, George Opit, Radek Aulicky, Yang Cao, Fu-Jun Li, Yi Wu, Tao Zhang, Zhi-Hong Li

**Affiliations:** 1 Department of Entomology, College of Plant Protection, China Agricultural University, Beijing, 100193 China; 2 Crop Research Institute, Drnovská 507, 161 06 Prague 6, Czech Republic; 3 Department of Entomology and Plant Pathology, 127 Noble Research Center, Oklahoma State University, Stillwater, OK 74078 USA; 4 Academy of State Administration of Grain, Beijing, 100037 China

## Abstract

Stored-product psocids (Psocoptera: Liposcelididae) are cosmopolitan storage pests that can damage stored products and cause serious economic loss. However, because of the body size (~1 mm) of eggs, nymphs, and adults, morphological identification of most stored-product psocids is difficult and hampers effective identification. In this study, 10 economically important stored-product *Liposcelis* spp. psocids (*Liposcelis brunnea, L. entomophila, L. decolor, L. pearmani*, *L. rufa, L.mendax*, *L. bostrychophila*, *L. corrodens*, *L. paeta, and L. tricolor*) were collected from 25 geographic locations in 3 countries (China, Czech Republic, and the United States). Ten species-specific probes for identifying these 10 psocid species were designed based on ITS2 sequences. The microarray method and reaction system were optimized. Specificity of each of the ten probes was tested, and all probes were found suitable for use in identification of the respective10 *Liposcelis spp*. psocids at 66 °C. This method was also used to identify an unknown psocid species collected in Taian, China. This work has contributed to the development of a molecular identification method for stored-product psocids, and can provide technical support not only to facilitate identification of intercepted samples in relation to plant quarantine, but also for use in insect pest monitoring.

## Introduction

Stored-product psocids (Psocoptera: Liposcelididae), also called booklice, barklice, or dustlice, belong to the genus *Liposcelis* and are regarded as worldwide storage pests^1^. This pest infests stored products, such as cereal grains and their processed products, other non-cereal-based foods, books, records and biological specimens, and high population densities cause serious economic losses^2^. There are currently, 126 species of stored-product psocids identified worldwide and these have been placed in 2 sections (Sections I and II) and 4 groups (Groups IA, IB, IIC and IID). Among them, 10 species, *Liposcelis brunnea* (Motschulshy), *L. entomophila* (Enderlein), *L. decolor* (Pearman), *L. pearmani* (Lienhard), *L. rufa* (Broadhead), *L. mendax* (Pearman), *L. bostrychophila* (Badonnel), *L. corrodens* (Motschulsky), *L. paeta* (Pearman), and *L. tricolor* (Badonnel) are considered common species which are pests of substance and a threat to stored-product trade^3,4^. In the last 30 years psocids have risen to prominence as serious pests and not just nuisance pests. However, because of their small body size (~1 mm), they are difficult to identify using morphological characteristics; this hampers fast identification of the various psocid species using any of the life stages of these pests^5^. The frequency of interception of stored-product psocids at ports of entry is currently approximately 2,300 times/year based on data for 2014–2016, and the frequency of these interceptions continue to increase with increase in international trade by China (based on the intercept and capture data provided by Chinese Academy of Inspection and Quarantine. Unpublished). In addition, this pest can also be found in many kinds of goods (commodities) and packaging materials^6^. Therefore, accurate and fast identification methods are urgently needed for those working in plant quarantine, grain reposition and transportation, and archives management.

In order to overcome the limitations of morphological identification methods, many molecular technologies, including restriction fragment length polymorphism (PCR-RFLP)^3^, DNA barcoding^4^, species-specific primer PCR (SS-PCR)^7,8^, multiplex endpoint PCR and multiplex TaqMan qPCR^9^, have been developed for psocid identification. Molecular markers are the foundation for molecular identification, and both mtDNA (mitochondrial DNA) and rDNA (ribosomal DNA) are species-specific and can be used as molecular markers for species identification^10,11^. To date, many molecular makers, including cytochrome c oxidase subunit I (*COI*), 16S ribosomal RNA gene (*16S rDNA*), internal transcribed spacer2 (*ITS2*) and microsatellites have been confirmed effective for identifying stored-product psocids from different species^12^. Successful use of these molecular markers and methods has made stored-product psocid identification much quicker and more accurate, also laying a foundation for establishing a systemic and rapid molecular identification system for this small pest.

Among numerous molecular methods, the microarray methodology has received much attention; it has been extensively researched and has practical applications, stemming from its advantages of high throughput, high parallelism and high sensitivity. The solid gene chip, as a traditional microarray method, has been widely used in many areas including disease diagnosis and prediction, drug screening, agriculture, food hygiene and environmental monitoring^13,14^. In recent years, this method is beginning to be used in insect identification. Sequences of two mitochondrial genes (*COI* and *ND2*) and a ribosomal gene (*ITS2*) have been used to design species-specific probes, and a gene chip method thus developed was able to provide simultaneous identification of nine important medical and veterinary species, including immatures, from genera of *Aedes*, *Anopheles*, *Armigeres*, and *Culex* ^15^. The gene chip helps overcome limitations caused by phenotypic variations in adults, lack of recognizable features in immatures, and the fragility of mosquitoes^15^. An innovative and accurate rapid high-throughput microfluidic dynamic array method based on COI DNA sequence has also been used for detecting 27 economically important tephritidae species in six genera (*Anastrepha*, *Bactrocera*, *Carpomya*, *Ceratitis*, *Dacus* and *Rhagoletis*)^16^. A gene chip based on the *COI* gene has been constructed for identifying 3 species of quarantine *Ceratitis* (*C. capitata*, *C. cosyra* and *C. rosa*), 3 species of *Frankliniella*, and 15 species of 5 genera in Culicidae^17–19^.

Although the gene chip has been used to identify some kinds of insects, there is currently no report of this method being applied in stored-product psocid identification. In this study, for the first time we designed 10 species-specific probes for identifying 10 stored-product psocid species, tested the specificity of these probes, established a microarray method based on the ITS2 rDNA sequence, and then optimized the method and reaction system. This method and the species-specific probes were also used to identify an unknown psocid species that was collected in China. Our results showed that the microarray method using species-specific probes based on ITS2 rDNA sequence can improve the molecular identification of stored product psocid species and can increase the likelihood for correctly identifying an intercepted sample in the field, in relation to plant quarantine. It can also be used to facilitate insect pest monitoring in the field of stored product protection.

## Results

### Probe sequences for ten species

All the probes used in this research were designed based on ITS2 sequences (Fig. S1). The species-specific probes that were used for the identification of ten booklice species (Table 1) are listed in Table 2. Quality-control probes, including the anchor position probe, positive probe, negative probe and blank control probe are listed in Table 3, all the probes were oligonucleotide fragments with no sequence homology to ITS2 sequences of the ten species. The anchor position probe was used for detecting whether there was a normal coupling between the probe and the chip, because there was a Cy5 label at the 5′ end of this kind of probe. In addition, this kind of probe can also be used as a reference for positioning other probes when reading the hybridization result. The positive probe was used as a positive control for detecting whether the hybridization was successful or not. The negative control can monitor if there is non-specific hybridization.

**Table 1:** *Liposcelis* species and populations used in this study.

Species	Group	Populations	Collection regions
*Liposcelis brunnea*	IA	*L. brunnea*_P-CZ	Prague, Czech Republic
		*L. brunnea*_USA	USA
*L. entomophila*	IA	*L. entomophila*_BJ- P. R. China	Beijing, China
		*L. entomophila*_HBWH- P. R. China	Wuhan, Hubei Province, China
		*L. entomophila*_P-CZ	Prague, Czech Republic
		*L. entomophila*_CQ- P. R. China	Chongqing, China
*L. decolor*	IB	*L. decolor*_CQ- P. R. China	Chongqing, China
		*L. decolor*_P-CZ	Prague, Czech Republic
		*L. decolor*_USA	USA
*L*. *pearmani*	IB	*L. pearmani*_USA	USA
*L*. *rufa*	IB	*L. rufa*_USA	USA
*L*. *mendax*	IIC	*L. mendax*_JS-P. R. China	Jiangsu Province, China
*L*. *bostrychophila*	IID	*L. bostrychophila*_BJ- P. R. China	Beijing, China
		*L. bostrychophila*_GX- P. R. China	Guangxi, China
		*L. bostrychophila*_CQ- P. R. China	Chongqing, China
		*L. bostrychophila*_P-CZ	Prague, Czech Republic
		*L. bostrychophila*_USA	Manhattan, USA
*L*. *corrodens*	IID	*L. corrodens*_P-CZ	Prague, Czech Republic
		*L. corrodens*_USA	USA
*L*. *paeta*	IID	*L. paeta*_USA	USA
		*L. paeta*_HBSJZ-P. R.China	Shijiazhuang, Hebei Province, China
		*L. paeta*_ZJ-P. R.China	Zhejiang Province, China
		*L. paeta*_HBWH-P. R.China	Wuhan, Hubei Province, China
		*L. paeta*_ P-CZ	Prague, Czech Republic
*L*. *tricolor*	IID	*L. tricolor*_SD-P. R. China	Heze, Shandong Province, China

**Table 2:** Detection probes of gene chip for 10 species of *Liposcelis*.

Probe name	Species	Probe sequence (5′-3′)^a^	Length (bp)	Position (bp)
Lbr	*Liposcelis brunnea*	NH_2_-(T)_15_-AGGATCGAAGAAGGTCTTCCTCTC	24	394–417
Len	*L. entomophila*	NH_2_-(T)_15_-GAAGTAAGTCGATTTCCGAAACGTA	25	123–147
Lde	*L. decolor*	NH_2_-(T)_15_-GATAACAAGCATGCCCTAAGCAC	23	256–278
Lpe	*L*. *pearmani*	NH_2_-(T)_15_-TTCCTCGTCACGTTAGTCAGTTTG	24	304–327
Lru	*L*. *rufa*	NH_2_-(T)_15_-GGAAGAAGAACACTATAGAGAACGAT	26	186–211
Lme	*L*. *mendax*	NH_2_-(T)_15_-CTTCGGCACGAATAATGTGGAG	22	106–127
Lbo	*L*. *bostrychophila*	NH_2_-(T)_15_-CTGTGGAAGTGTCGAAAGATTTGAG	25	118–142
Lco	*L*. *corrodens*	NH_2_-(T)_15_-TGCAAAAACGGTTTCTCTGCGT	22	240–261
Lpa	*L*. *paeta*	NH_2_-(T)_15_-GTTTACCGACGATTTTGAGAGTGTC	25	98–122
Ltr	*L*. *tricolor*	NH_2_-(T)_15_-GAAAGAGAATGTCTCAGTAAATGGT	25	184–208

^a^All the probes had -NH_2_-(T)_15_- added at the 5′ side.

**Table 3:** Quality-control probes of gene chip for 10 species of *Liposcelis*

Probe Type	Code	Probe sequence (5′-3′)^a^
Anchor point probe	A	Cy5-GTCTTGTCTGATCTGAG-NH_2_
Positive probe	P	NH_2_-(T)_15_-GGTGACGGGGAATCAGGGTTCGATT
Negative probe	N	NH_2_-(T)_15_-GACTATAGTATAAGCGCGGTCCA
Blank control	B	

^a^The anchor point probe had -Cy5 added at the 5′ side and -NH_2_ added at the 3′ side, while the other probes had -NH_2_-(T)_15_- added at the 5′ side.

### Microarray chip design

**Figure 1:**
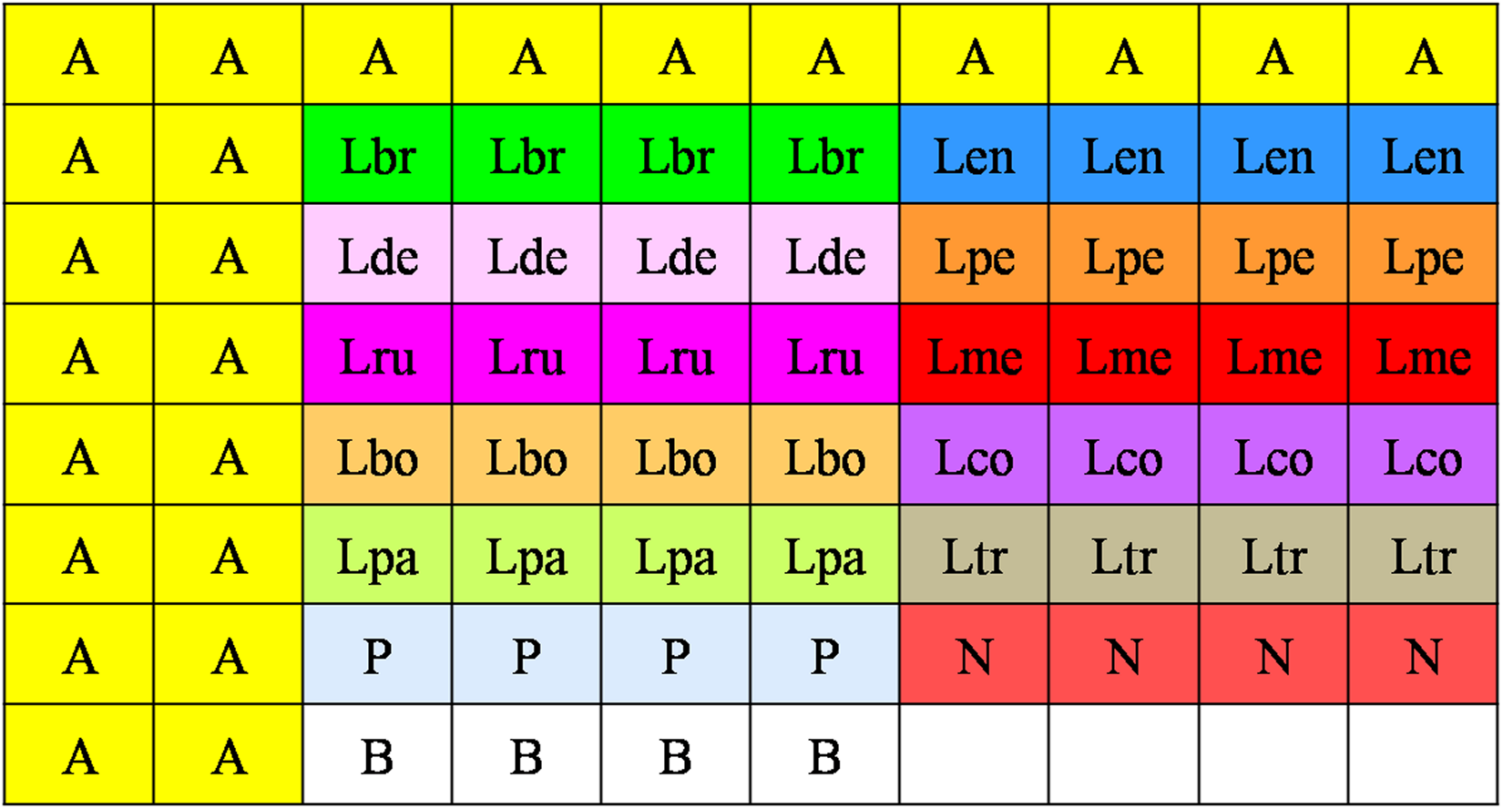
Layout of gene chip probes for 10 species of *Liposcelis*. The abbreviations and different colors in the table represent different kinds of probes. A: Anchor point probe; P: Positive control probe; N: Negative control probe; B: Blank control; Lbr: Probe for *Liposcelis. brunnea*; Len: Probe for *L. entomophila*; Lde: Probe for *L. decolor*; Lpe: Probe for *L*. *pearmani*; Lru: Probe for *L*. *rufa*; Lme: Probe for *L*. *mendax*; Lbo: Probe for *L*. *bostrychophila*; Lco: Probe for *L*. *corrodens*; Lpa: Probe for *L*. *paeta*; Ltr: Probe for *L*. *tricolor*.

There were 12 of the same probe lattices, which distributed as 6 rows and 2 columns for each microarray chip. The detailed layout of the probes in each probe lattice is shown in Fig. 1. There were 8 rows and 10 columns for each probe lattice, and the first row, the first and second column were the anchor position probes. The other points were for the species-specific probes and there were four repeats for each probe. The space between columns was 0.4 mm, while the space between rows was 0.8 mm. This probe lattice can be used for identifying ten species of stored-product psocids.

### Establishment of the reaction system

On the basis of optimizing primer concentrations, upstream and downstream primer proportion, detection sensitivity and hybridization temperature, one optimized reaction system was established for the microarray chip identification method. Asymmetric PCR was useful for amplifying enough ITS2 sequences, especially when the proportion of upstream and downstream primers is 1:20 (Fig. S2). The best result, comprising the highest hybridization efficiency, the best detection specificity and the weakest false positive can be obtained at the temperature of 66 °C (Fig. S3). The detection sensitivity showed that the signal was strongest when DNA without dilution was used as the template for ITS2 sequence amplification (Fig. S4).

### Microarray scanning results for the 10 species of *Liposcelis*

The hybridized pattern was scanned at the excitation wavelength of 635 nm/532 nm, and the Cy5 displayed red color at this wavelength. The signal value for each hybridized site was obtained and used as the basis for deciding whether the hybridization was successful or not, whether the hybridization was positive or not, and whether there are some problems of hybridization. The value of F635 Mean-B635 (the mean spot pixel intensity at wavelength 635 nm/532 nm with the background subtracted) was used as the signal value. The signal value for the blank and negative control was the mean value of four repeated hybridized sites. If the signal value for one detecting site minus the mean signal value of blank control was higher than ten times the mean signal value of negative control, and also higher than 500, this detecting site was determined as positive, which meant that the probe for this detecting site hybridized with the provided DNA successfully. When the mean signal value of four repeated detecting sites for each probe was positive, it means the hybridization result for this probe was positive. The result was inaccurate under certain circumstances — when the negative hybridization result for some or all the anchor point probes indicated the probe had not combined with the chip, a positive hybridization result for negative control indicated there was non-specific hybridization or there was pollution during the process of amplification and hybridization, while a negative hybridization result for positive control indicated a problem existing in the process of amplification or hybridization. When the hybridization result for the positive probe and one specific probe was positive, it implies the microarray worked well and showed there was successful hybridization between the specific probe and the corresponding *Liposcelis* species.

**Figure 2:**
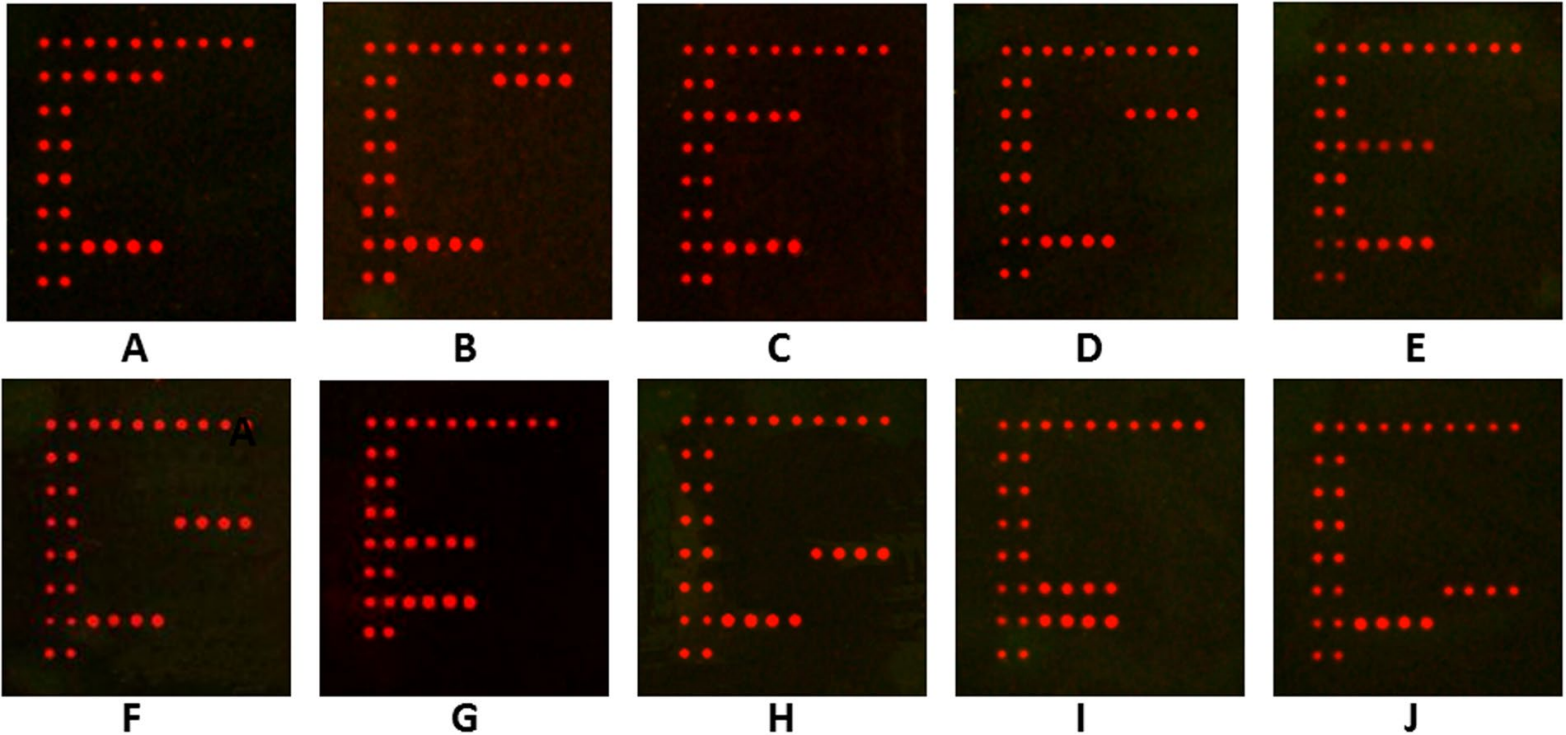
Microarray scanning results for the 10 species of *Liposcelis*. (**A**) Scanning result for hybridization with the ITS2 sequence from *L. brunnea*, which were collected from Prague in Czech Republic or the United states (USA). (**B**) Scanning result for hybridization with the ITS2 sequence from *L. entomophila*, which were collected from Beijing, Chongqing, Wuhan (Hubei Province) in China, or Prague in Czech Republic. (**C**) Scanning result for hybridization with the ITS2 sequence from *L. decolor*, which were collected from Chongqing in China, Prague in Czech Republic or the USA. (**D**) Scanning result for hybridization with the ITS2 sequence from *L. pearmani*, which were collected from the USA. (**E**) Scanning result for hybridization with the ITS2 sequence from *L. rufa*, which were collected from the USA. (**F)** Scanning result for hybridization with the ITS2 sequence from *L. mendax*, which were collected from Jiangsu Province in China. (**G**) Scanning result for hybridization with the ITS2 sequence from *L. bostrychophila*, which were collected from Beijing, Guangxi, Chongqing in China, Prague in Czech Republic, or Manhattan in the USA. (**H**) Scanning result for hybridization with the ITS2 sequence from *L. corrodens*, which were collected from Prague in Czech Republic, or the USA. (**I**) Scanning result for hybridization with the ITS2 sequence from *L. paeta*, which were collected from Shijiazhuang (Hebei Province), Zhejiang Province, Wuhan (Hubei Province) in China, Prague in Czech Republic, or the USA. (**J**) Scanning result for hybridization with the ITS2 sequence from *L. tricolor*, which were collected from Heze (Shandong Province) in China.

According to this analysis method, the hybridization results for ten species-specific probes are shown in Fig. 2. Figure 2A shows a successful hybridization between probe Lbr and the ITS2 sequence from *L. brunnea*, which were collected from the United States (USA) or Prague in Czech Republic. Figure 2B shows the scanning result for hybridization between probe Len and the ITS2 sequence from *L. entomophila*, which were collected from Beijing, Chongqing, Wuhan (Hubei Province) in China, or Prague in Czech Republic. Figure 2C shows a successful hybridization between probe Lde and the ITS2 sequence from *L. decolor*, which were collected from Chongqing in China, Prague in Czech Republic or the USA. Figure 2D shows that probe Lpe can hybridize successfully with the ITS2 sequence from *L. pearmani*, which were collected from the USA. The successful hybridization between probe Lru and the ITS2 sequence from *L. rufa* (USA population) is shown in Fig. 2E. The scanning result for hybridization between probe Lme and the ITS2 sequence from *L. mendax*, which were collected from Jiangsu Province in China, is shown in Fig. 2F. As shown in Fig. 2G, probe Lbo can hybridize successfully with the ITS2 sequence from *L. bostrychophila*, which were collected from Beijing, Guangxi, Chongqing in China, Prague in Czech Republic, or Manhattan in the USA. *L. corrodens* ITS2 sequence from two populations, including Prague in Czech Republic and the USA, can hybridize successfully with probe Lco separately, and the scanning result is shown in Fig. 2H. Probe Lpa can hybridize successfully with the ITS2 sequence from *L. paeta*, which were collected from Shijiazhuang (Hebei Province), Zhejiang Province, Wuhan (Hubei Province) in China, Prague in Czech Republic, or the USA (Fig. 2I). Figure 2J shows a successful hybridization between probe Ltr and the ITS2 sequence from *L. tricolor*, which were collected from Heze (Shandong Province) in China.

### Practice of the microarray chip method

**Figure 3:**
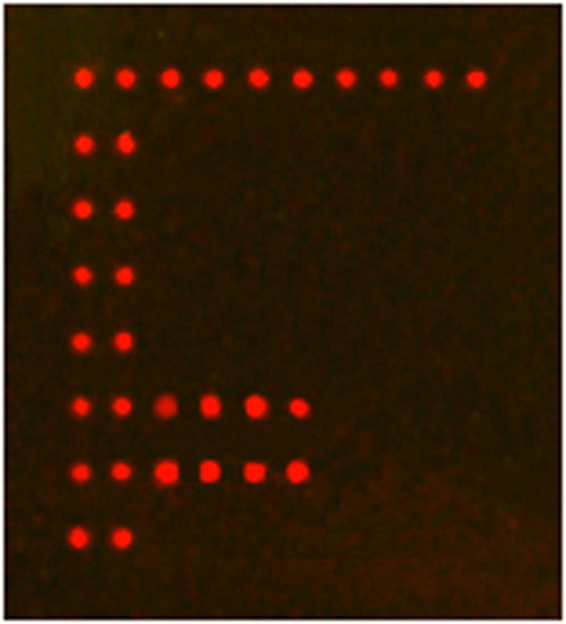
Application of this gene chip method for identifying one unknown *Liposcelis* species collected from Taian (Shandong Province). Scanning result shows that this unknown species is *L. paeta*.

To examine the specificity of these 10 species-specific probes and the microarray method in practice, a sample of one *Liposcelis* species, which was collected from Taian, Shandong Province in China, was used for DNA extraction and ITS2 sequence amplification. As shown in Fig. 3, after being hybridized with the provided ITS2 sequence, the microarray showed positive signals at the position of probe Lpa and the positive probe, which is the same as in Fig. 2I. The scanning result indicated that the *Liposcelis* species from Taian, Shandong Province was *L. paeta*. The traditional morphological identification result also showed that the supplied sample had the typical morphological characteristics of *L. paeta*, including tawny body color, segmacoria at the posterior edge of the third and fourth abdomen tergum (link type abdomen), one pair of bristles at the latter part of antesternum, no furcation at the end of gonapophysis, short and tapered receptor on the maxillae palpus, three ommatidia and so on. ITS2 sequence of this unknown species was also amplified and sequenced. The sequence was submitted to NCBI and got a GenBank NO (MF434638). NCBI blast result also showed that the unknown species has a highest similarity with *L. paeta*. The microarray result was coincident with the morphological identification and NCBI blast result.

## Discussion

In this study, 10 species-specific probes based on ITS2 rDNA were provided and an identification method using a microarray chip was developed. An optimized reaction system that comprised primer concentrations, upstream and downstream primer proportion, detecting sensitivity and hybridization temperature was established. This method was used to identify 10 economically important *Liposcelis* species from 25 geographical populations in one microarray chip. It was also used to identify an unknown psocid species that was collected from Taian, China. To the best of our knowledge, although ITS2 rDNA has been used as an effective molecular marker for identifying stored-product psocids for a long time, this is the first report of using species-specific probes based on ITS2 rDNA for psocid species identification.

For eukaryotes, there are two internal transcribed spacers (ITS1 and ITS2), highly repetitive sequences that are removed after DNA transcription^20,21^. ITS1 is located between 18S rDNA and 5.8S rDNA, whereas ITS2 is located between 5.8S rDNA and 28S rDNA^22,23^. As with 5.8S rDNA, 18S rDNA, and 28S rDNA, ITS2 displays high conservation within a species but significant variation among species; this characteristic makes this gene suitable for use as a molecular marker for identification and phylogenetic analysis in many species^22,24,25^. In recent years, ITS2 is increasingly being used in taxonomy, phylogenetic development, and investigating species evolution^26,27^. Since 2011, the ITS gene has been used to study the gene structure and phylogenetics of stored-product psocid species. Wei *et al*.^6^ (2011) compared the differences of ITS (ITS1-5.8S-ITS2) sequence among six stored-product psocid species (*L. bostrychophila, L. entomophila, L. decolor, L. paeta, L. tricolor, and L. yunnaniensis*) collected from 16 geographical locations, provided six pairs of primers for amplifying the ITS2 region of the six species, and then constructed a species-specific primer identification method based on multiple PCR. Using a larger number of geographic populations, Zhao *et al*. (2016) provided 10 pairs of species-specific primers based on ITS2 rDNA for identifying 10 economically important stored-product psocid species (*L. bostrychophila, L. entomophila, L. decolor, L. paeta, L. brunnea, L. corrodens, L. mendax, L. rufa, L. pearmani*, and *L. tricolor*) from 35 geographic populations in 5 countries (China, Czech Republic, Denmark, Germany, and the United States). Our study used the 53 ITS2 sequences submitted by Li *et al*. for probe design, and then constructed a microarray method to identify 10 psocid species from 25 geographic locations for the first time. The result indicated that ITS2 rDNA gene-based species-specific probes can be used for species identification.

Because primer and probes specificity are the most important factors for species identification, it means higher annealing temperature can enhance the specificity. The microarray used a higher annealing temperature (66 °C) than the previous identification methods for psocid, especially the species-specific primer method published by Zhao *et al*. in 2016 (52 °C). Additionally, the microarray method can also improve the specificity by two steps, including asymmetric PCR and the subsequent hybridization. In published studies, it’s rare to find a positive control in PCR-based methods for identification, because of how difficult it is for this to occur. However, in this study, we set a positive probe which can indicate normal hybridization and this become of obvious advantage for microarray. In the present study, 2.5 h were required to identify 12 psocid samples on one chip with high efficiency (excluding DNA extraction); the sequencing-based method used in the identification of 14 Noctuoidea species using a thin-film biosensor required 4 h to identify 14 Noctuoidea species^28^. The throughput can also be increased by dotting more probes in one layout. When identifying one unknown species using the PCR-based method, 10 reaction volumes (total 250 μL) for 10 pairs of species-specific primers were used. While the microarray-based method requires only one reaction volume (25 μL) for 10 pairs of species-specific probes. Thus, the conclusion is that this study developed a reagent-saving identification method with high specificity, accuracy and efficiency. This technique also offers great potential for detecting high species diversity, and can satisfy plant quarantine needs for rapid, accurate and effective identification.

Although the microarray method has some advantages, it sometimes produces false positive results. For further research, more species from more populations should be used to increase the specificity of probes. The optimal reaction volume and temperature, optimal distribution of probes in the microarray chip, optimal concentration of DNA and other protocol details need to be improved to avoid the possible false positive results. Additionally, only adults were used for this study, but future research needs to use other stages as well. DNA was extracted from a single adult using a DNA extracting Kit. However, typically, eggs, nymphs and body parts are intercepted and rarely are adult individuals collected. The DNA extraction method used in this study can also be applicable for eggs and nymphs. The microarray identification method developed was not used for non-adult samples hence this needs to be the subject of future studies.

As previously mentioned, stored-product psocids are now recognized as cosmopolitan pests of substance^6,29–31^. The economic losses, high interception frequency, and difficulty of morphological identification caused by their small body size necessitate the development of rapid molecular identification methods. Many evidences have shown that even closely related *Liposcelis* species that are hard to differentiate by morphological methods may respond differentially to various control methods and to cereal storage environmental conditions. For example, Guedes *et al*. (2008a) found that *L. bostrychophila* was more tolerant than *L. entomophila* in relation to response to surfaces treated using ß-cyfluthrin and chlorfenapyr insecticides; this was due to differential species-specific behavioural reaction to pesticide residues^32^. Guedes *et al*. (2008b) showed that *L. entomophila* was twice as tolerant to heat treatment as *L. reticulatus* ^33^. In addition, various *Liposcelis* species may exhibit pose different levels of risk based on variation in how rapidly their populations grow after infestation under identical storage temperature and humidity^34–36^. Thus, correctly identifying psocid species responsible for infestations is important in selecting effective control methods; developing rapid, systematic molecular identification methods for stored-product psocid identification facilitates their control^12,37^. The microarray methodology, which is an accurate, high throughput, and convenient molecular identification method, has played an important part in improving the timeliness of psocid identification for the port and food sector in China. Future effort needs to develop commercial detection technology and products, partly through pilot promotion in different ports. Improving the specification and optimization of the selective reaction system can help this method (microarray method) play an increasingly important role in facilitating quarantine and stored-product protection in China and elsewhere.

## Methods

### Sample collection and morphological identification

Ten stored-product psocid species from 25 geographic populations were collected from locations in China, Czech Republic and the United States. All the 25 geographic populations were identified by two taxonomists Drs. Zhihong Li and Zuzana Kučerová, using morphological characteristics, and were preserved in absolute ethyl alcohol at the Plant Quarantine and Invasion Biology Laboratory of China Agricultural University (Table 1).

### DNA extraction

Total DNA of 10 psocid species was extracted from individual booklice (Table 1) using a Tiangen DNA Extraction Kit with instructions modified for booklice. The extraction process is the same as that described by Zhao *et al*. in 2016^8^. The DNA was stored at −20 °C.

### ITS2 sequence amplification

Partial sequence of human gene (KY962518.1, from 4019 to 4136) was used for positive control, and this sequence can be amplified by using one pair of primers, For2 and Rev2 (Table 4) via normal symmetric PCR.One pair of universal primers, For1 and Rev1 (Table 4), used by Zhao *et al*., were used for amplifying the ITS2 gene of 10 stored-product psocid species based on a screening process. Oligo synthesis was conducted by the Beijing Aoke Biotechnology Company. An asymmetric PCR was performed using the 5′ Cy5-labelled reverse primer (Cy5-Rev1) at a concentration 19 times higher than that of the forward primer (For1), which means the proportion of the two primers is 20:1. A 25 μL PCR system was used, which contained 1.5 μL of DNA template (psocid DNA or positive control amplification product, 25 ng/μL), 2.5 μL of 10× EX *Taq* Buffer, 2 μL of dNTP Mixture (2.5 mM), 0.2 μL EX *Taq*, 0.5 μL of forward primer (For1, 1 μM), 1 μL of reverse primer (Cy5-Rev1, 10 μM), and 17.3 μL of ddH_2_O. The PCR programming conditions: a 4 min DNA pre-denaturation at 94 °C and 35 amplification cycles (30 s for denaturation at 94 °C, 30 s for DNA annealing at 52 °C, and 1 min for extension at 72 °C) with a final extension at 72 °C for 10 min.

**Table 4:** Universal primers for ITS2 amplification and long primers for positive template amplification.

Primer name	Primer sequence
FOR1	TGTGAACTGCAGGACACATG
REV1	GTCTTGTCTGATCTGAG
Cy5-REV1	Cy5-GTCTTGTCTGATCTGAG
FOR2	TGTGAACTGCAGGACACATGCAACTTTCGATGGTAGTCGCCG
REV2	GTCTTGTCTGATCTGAGGCCTGCTGCCTTCCTTGGATG

### Probe design

ITS2 sequences of the 25 populations of 10 *Liposcelis* species used were retrieved from GenBank and were aligned using DNAMAN 6.0; species-specific regions suitable for probe design were selected for species-specific probe design. Primer premier 5.0 and DNASTAR Laser gene 7.0 were used to design the probes. Quality-control probes, including anchor point probe, positive probe, negative probe and blank control probe, were designed to confirm whether the PCR and the hybridization were normal.

### Microarray chip construction

On a commercially purchased aldehyde modified glass slide (Capital Bio Corporation, China), the probe solution (50 μM), prepared by mixing the 100 μM oligonucleotide probe and 2× probe solution, was spotted on the surface of polymer membrane using the DNA Microarray and Protein Microarray Spotter AD1500 (Biodot, the USA), and four spots were used for each booklice species. These spotted slides were then incubated at 37 °C in a humid chamber for ≥12 h. The slides were then submerged in wash buffer (300 mM Bicine, 300 mM NaCl, 0.1% SDS) for 30 min at 66 °C, then in sterile deionized water for 5 min and dried by centrifugation. Before hybridization, fencing was pasted to divide the glass slides into hybridization plots. And then the layout was covered to make it separate from the others, so that the reactions would not influence each other.

### Microarray hybridization and scanning

Ten μL of mixture containing 4.5 μL of the labeled PCR product of booklice species, 1 μL of applied positive sequence, and 4.5 μL of hybridization buffer were added to the plots on the microarray, which was put in a hybridization box with water to create wet conditions. The microarray was then incubated at 66 °C in the oven with vibration for 2.5 h. After removing the fencing, the glass slides were washed in wash buffer I (2× SSC, 0.1% SDS) two times for 5 min, then in wash buffer II (0.2× SSC, 0.1% SDS) two times for 5 min, and the last washing was conducted once in wash buffer III (0.2× SSC). Slides were put in absolute ethyl alcohol for extraction and dried after washing. The hybridization pattern was detected using a Microarray Scanner InnoScan 900 (Innopsys, France), and the software Mapix 2.9.5 was used to get the fluorescence signal data. For the species with more than 2 geographic populations, at least 2 adults from each population were used for hybridization; while for the species with only one geographic population, at least 3 adults were used for probes detecting. At least 3 biological replicates were used for each probes detector. All the experiments were repeated for three times.

### Application of the microarray chip method

The microarray chip method and the 10 species-specific probes were used to identify one unknown stored-product psocid species from Taian, Shandong Province, China. Three adults were used for the biological replicates. Meanwhile, the traditional morphological identification and Sanger sequencing method was also used to identify this species to verify the result of the microarray method.

## Electronic supplementary material

Dataset 1
