# Supplementary material for: Molecular Identification of ten species of stored-product psocids through microarray method based on ITS2 rDNA
Source: Sci Rep. 2017 Dec 1;7:16694. doi: 10.1038/s41598-017-16888-z (PMC5711863; doi:10.1038/s41598-017-16888-z)
Supplement: Supplementary file 1 — Dataset 1 [file 41598_2017_16888_MOESM1_ESM.doc]

**Molecular Identification of ten species of stored-product psocid****s through microarray method based on ITS2 rDNA**

**Li-Jun Liu^1,*^, Ao-Han Pang^1,*^, Shi-Qian Feng^1^, Bing-Yi Cui^1^, Zi-Hua Zhao^1^, Zuzana Kučerová^2^, Václav Stejskal^2^, George Opit^3^, Radek Aulicky^2^, Yang Cao^4^, Fu-Jun Li^4^, Yi Wu^4^, Tao Zhang^1,4^, Zhi-Hong Li^1^**

^1^Department of Entomology, College of Plant Protection, China Agricultural University, Beijing 100193, China.

^2^Crop Research Institute, Drnovská 507, 161 06 Prague 6, Czech Republic.

^3^Department of Entomology and Plant Pathology, 127 Noble Research Center, Oklahoma State University, Stillwater, OK 74078, USA.

^4^Academy of State Administration of Grain, Beijing 100037, China.

*These authors contributed equally to this work. Correspondence and requests for materials should be addressed to Z.-H.L. (email: lizh@cau.edu.cn)

**Figure legends**

**S Figure 1. Similarity of 10 species of *Liposcelis* in the first 100bp of ITS2 rDNA.**

*L. tricolor-*Shandong: ITS2 sequence from *L. tricolor* of Shandong population. *L. mendax-*Jiangsu: ITS2 sequence from *L. mendax* of Jiangsu population. *L. rufa-*US: ITS2 sequence from *L. rufa* of the USA (United States of America) population. *L. brunnea -*US: ITS2 sequence from *L. brunnea* of the USA population. *L. corrodens -*US: ITS2 sequence from *L. corrodens* of the USA population. *L. paeta -*Taian: ITS2 sequence from *L. paeta* of Taian population. *L. decolor-*Chongqing: ITS2 sequence from *L. decolor* of Chongqing population. *L. bostrychophila -*Beijing: ITS2 sequence from *L. bostrychophila* of Beijing population. *L. entomophila-*Guangxi: ITS2 sequence from *L. entomophila* of Guangxi population.

**S Figure 2. Scanning results for hybridization between species-specific probe and the ITS2 sequence from asymmetric and symmetric PCR.** A: asymmetric PCR; B: symmetric PCR

**S Figure 3. Scanning results of different hybridization temperature.** A. 54℃. B. 58℃. C. 62℃. D. 66℃.

**S Figure 4.** **Scanning results for hybridization between species-specific probe and ITS2 sequence amplification product with different dilution ratios**. A: No dilution (0*); B: 5 times dilution (5*); C: 25 times dilution (25*); D: 125 times dilution (125*).

**S Figure 5. Images representing three replicates for each probe.** A. Layout of gene chip probes for ten species in supplementary materials. **B 1-3**: Scanning result for hybridization with the ITS2 sequence from *L. brunnea*, which were collected from Prague in Czech Republic or the United states (USA). **C1-3**: Scanning result for hybridization with the ITS2 sequence from *L. entomophila,* which were collected from Beijing, Chongqing, Wuhan (Hubei Province) in China, or Prague in Czech Republic. **D1-3**: Scanning result for hybridization with the ITS2 sequence from *L. decolor,* which were collected from Chongqing in China, Prague in Czech Republic or the USA. **E1-3**: Scanning result for hybridization with the ITS2 sequence from *L. pearmani,* which were collected from the USA. **F1-3**: Scanning result for hybridization with the ITS2 sequence from *L. rufa,* which were collected from the USA. **G1-3**: Scanning result for hybridization with the ITS2 sequence from *L. mendax,* which were collected from Jiangsu Province in China. **H1-3**: Scanning result for hybridization with the ITS2 sequence from *L. bostrychophila*, which were collected from Beijing, Guangxi, Chongqing in China, Prague in Czech Republic, or Manhattan in the USA. **I1-3**: Scanning result for hybridization with the ITS2 sequence from *L. corrodens*, which were collected from Prague in Czech Republic, or the USA. **J1-3**: Scanning result for hybridization with the ITS2 sequence from *L. paeta*, which were collected from Shijiazhuang (Hebei Province), Zhejiang Province, Wuhan (Hubei Province) in China, Prague in Czech Republic, or the USA. **K1-3**: Scanning result for hybridization with the ITS2 sequence from *L. tricolor*, which were collected from Heze (Shandong Province) in China. **L1-3**: Scanning result for hybridization with the ITS2 sequence from *L. paeta*, which were collected from Taian (Shandong Province) in China.


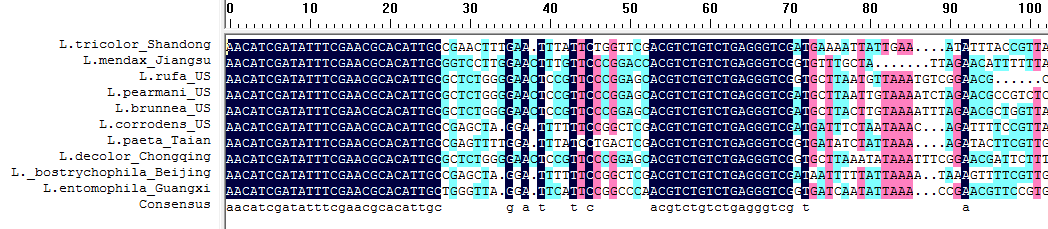


**S Figure 1. Similarity of 10 species of *Liposcelis* in the first 100bp of ITS2 rDNA**

*L. tricolor-*Shandong: ITS2 sequence from *L. tricolor* of Shandong population. *L. mendax-*Jiangsu: ITS2 sequence from *L. mendax* of Jiangsu population. *L. rufa-*US: ITS2 sequence from *L. rufa* of the USA (United States of America) population. *L. brunnea -*US: ITS2 sequence from *L. brunnea* of the USA population. *L. corrodens -*US: ITS2 sequence from *L. corrodens* of the USA population. *L. paeta -*Taian: ITS2 sequence from *L. paeta* of Taian population. *L. decolor-*Chongqing: ITS2 sequence from *L. decolor* of Chongqing population. *L. bostrychophila -*Beijing: ITS2 sequence from *L. bostrychophila* of Beijing population. *L. entomophila-*Guangxi: ITS2 sequence from *L. entomophila* of Guangxi population.


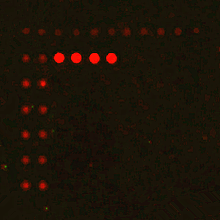

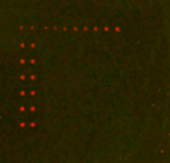


**A B**

**S Figure 2. Scanning results for hybridization between species-specific probe and the ITS2 sequence from asymmetric and symmetric PCR.**

A: asymmetric PCR; B: symmetric PCR


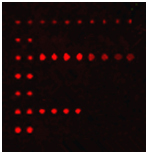

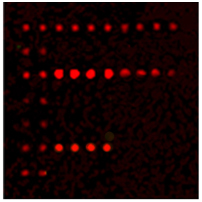

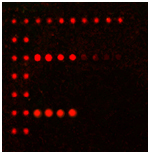

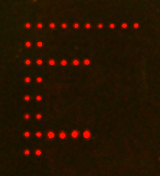


A B C D

**S Figure 3. Scanning results of different hybridization temperature**

A. 54 ◦C. B. 58 ◦C. C. 62 ◦C. D. 66 ◦C.


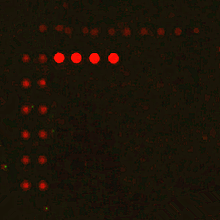

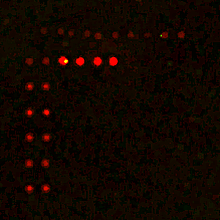

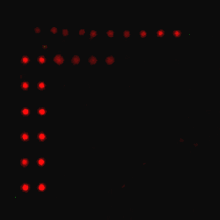

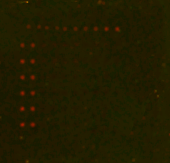


A B C D

**S Figure 4. Scanning results for hybridization between species-specific probe and ITS2 sequence amplification product with different dilution ratios**.

A: No dilution (0*); B: 5 times dilution (5*); C: 25 times dilution (25*); D: 125 times dilution (125*).

**
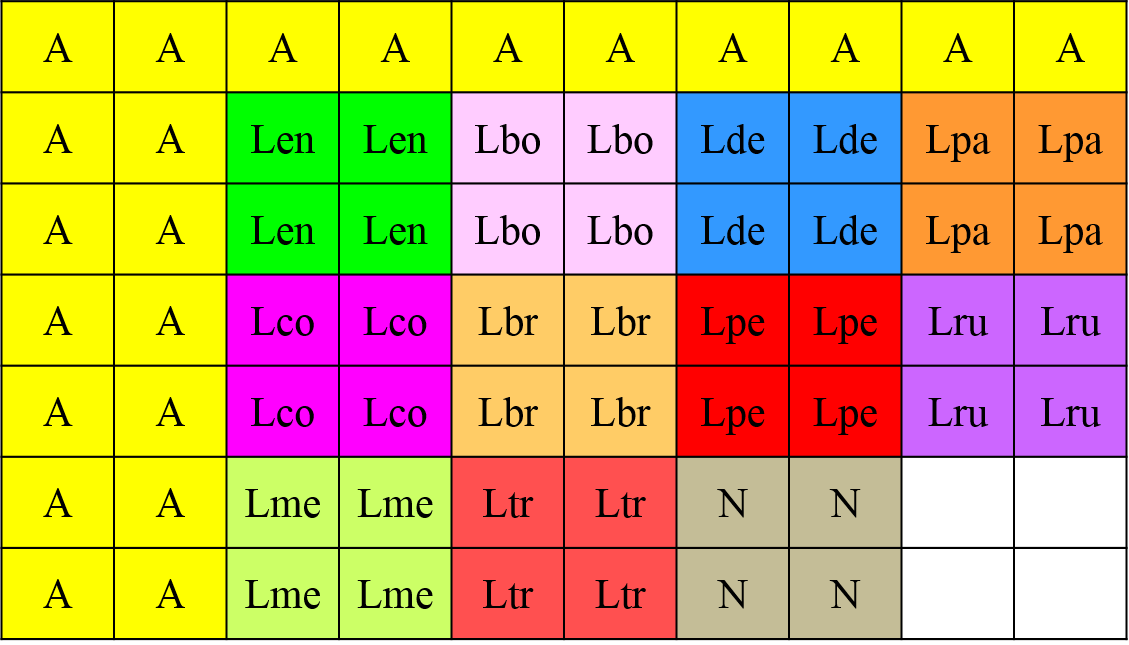
**

**A**

**
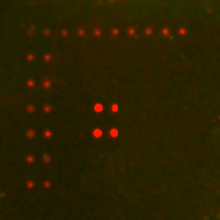
**  **
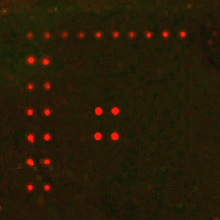

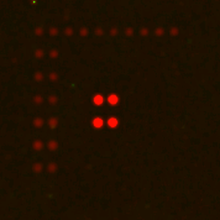
**

**B 1 2 3**

**
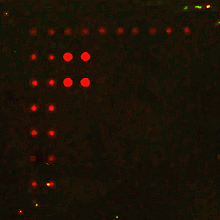

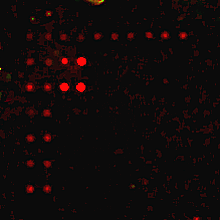

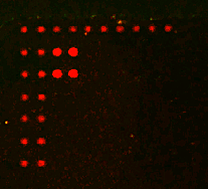
**

**C 1 2 3**

**
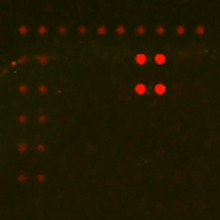

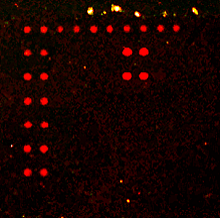

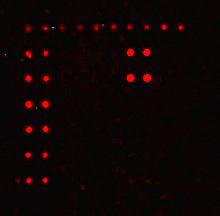
**

**D 1 2 3**

**
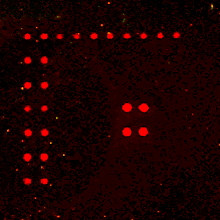

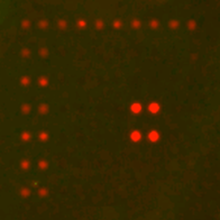

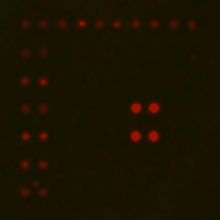
**

**E 1 2 3**

**
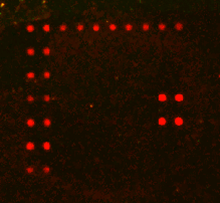

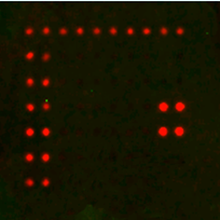

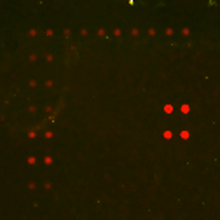
**

**F 1 2 3**

**
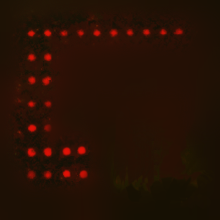

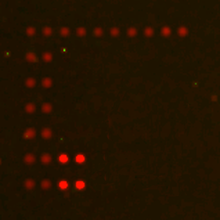

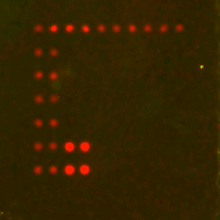
**

**G 1 2 3**

**
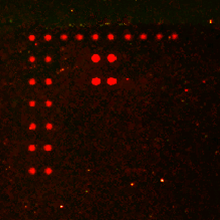

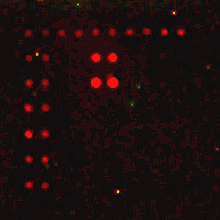

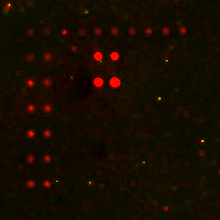
**

**H 1 2 3**

**
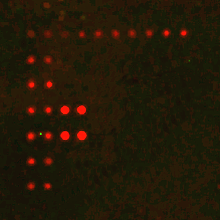

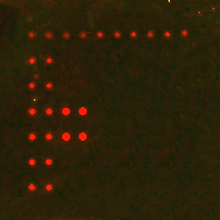

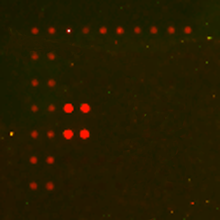
**

**I 1 2 3**

**
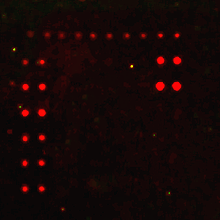

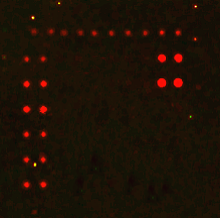

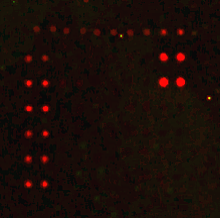
**

**J 1 2 3**

**
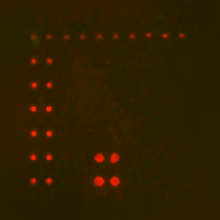

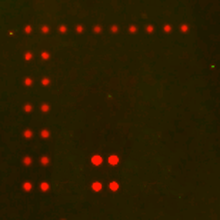

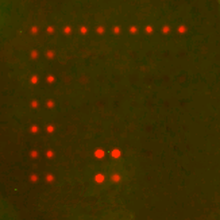
**

**K 1 2 3**

**
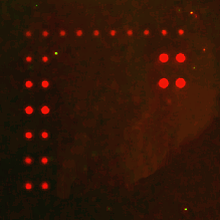

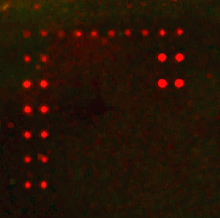

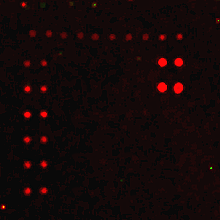
**

**L 1 2 3**

**S Figure 5. Images representing three replicates for each probe.** **A**. Layout of gene chip probes for ten species in supplementary materials. **B 1-3**: Scanning result for hybridization with the ITS2 sequence from *L. brunnea*, which were collected from Prague in Czech Republic or the United states (USA). **C1-3**: Scanning result for hybridization with the ITS2 sequence from *L. entomophila,* which were collected from Beijing, Chongqing, Wuhan (Hubei Province) in China, or Prague in Czech Republic. **D1-3**: Scanning result for hybridization with the ITS2 sequence from *L. decolor,* which were collected from Chongqing in China, Prague in Czech Republic or the USA. **E1-3**: Scanning result for hybridization with the ITS2 sequence from *L. pearmani,* which were collected from the USA. **F1-3**: Scanning result for hybridization with the ITS2 sequence from *L. rufa,* which were collected from the USA. **G1-3**: Scanning result for hybridization with the ITS2 sequence from *L. mendax,* which were collected from Jiangsu Province in China. **H1-3**: Scanning result for hybridization with the ITS2 sequence from *L. bostrychophila*, which were collected from Beijing, Guangxi, Chongqing in China, Prague in Czech Republic, or Manhattan in the USA. **I1-3**: Scanning result for hybridization with the ITS2 sequence from *L. corrodens*, which were collected from Prague in Czech Republic, or the USA. **J1-3**: Scanning result for hybridization with the ITS2 sequence from *L. paeta*, which were collected from Shijiazhuang (Hebei Province), Zhejiang Province, Wuhan (Hubei Province) in China, Prague in Czech Republic, or the USA. **K1-3**: Scanning result for hybridization with the ITS2 sequence from *L. tricolor*, which were collected from Heze (Shandong Province) in China. **L1-3**: Scanning result for hybridization with the ITS2 sequence from *L. paeta*, which were collected from Taian (Shandong Province) in China.
